# Supplementary material for: Viral Delivery of CAR Targets to Solid Tumors Enables Effective Cell Therapy
Source: Mol Ther Oncolytics. 2020 Apr 7;17:232–40. doi: 10.1016/j.omto.2020.03.018 (PMC7183102; doi:10.1016/j.omto.2020.03.018)
Supplement: Document S1. Figures S1–S4 [file mmc1.pdf]

OMTO, Volume *17*

## Supplemental Information

# Viral Delivery of CAR Targets to Solid Tumors Enables Effective Cell Therapy

**Amin Aalipour, Fabrice Le Boeuf, Matthew Tang, Surya Murty, Federico Simonetta, Alexander X. Lozano, Travis M. Shaffer, John C. Bell, and Sanjiv S. Gambhir**

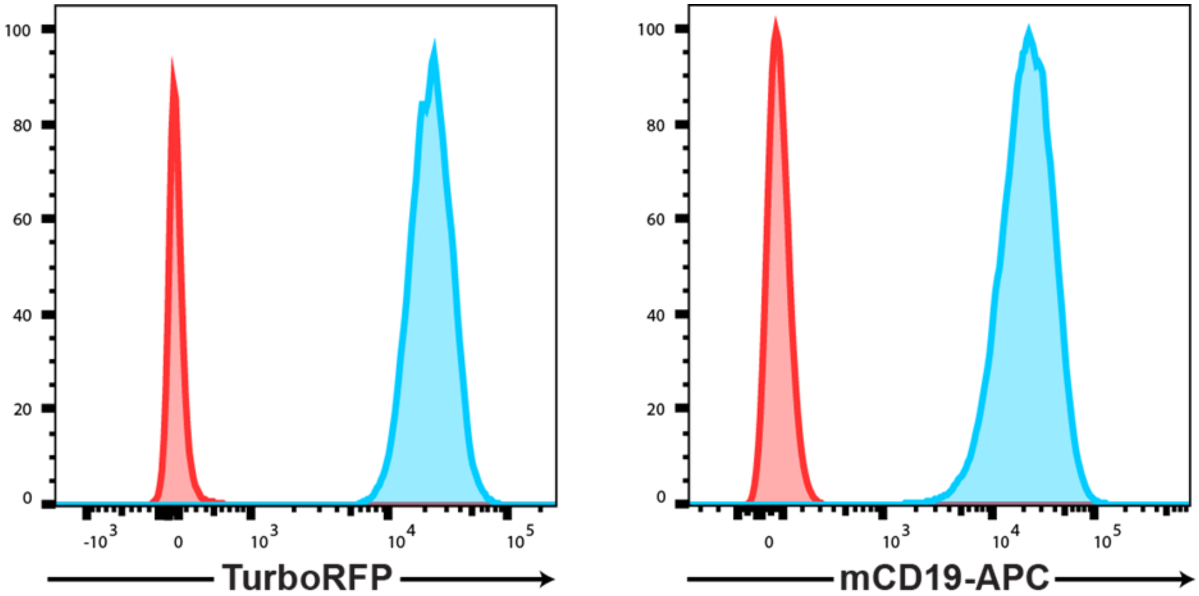


**Supplementary Figure S1. Generation of engineered B16 cell lines.**
Three cell lines were generated: B16-TurboRFP/RLuc8, B16-mCD19, and B16-TurboRFP/RLuc8-mCD19. Flow cytometry confirms uniform expression of TurboRFP (left) and mCD19 (right) transgenes. Data shown for B16-TurboRFP/RLuc8-mCD19 cell line (blue) relative to native B16 cell line (red).

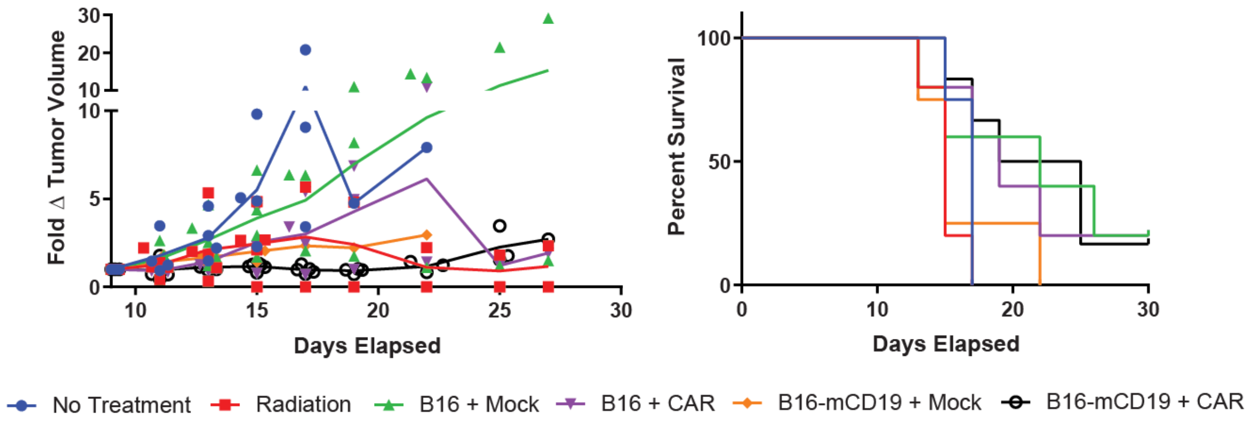


**Supplementary Figure S2. Intravenous delivery of mCD19 CAR T-cells is ineffective against B16 tumors constitutively expressing mCD19.** Intravenously delivered mCD19 CAR T-cells do not significantly delay B16-mCD19 tumor progression *in vivo* (left) or confer a survival benefit (right) relative to tumors treated with 5Gy TBI (radiation), mock T-cells, or antigen negative tumors treated with either mock or CAR T-cells. Kaplan-Meier survival curve p = 0.0902, df = 5, Chi square = 9.514 by Mantel-Cox test. Number of independent mice in each group as follows: n = 4 (No Treatment), n = 5 (Radiation), n = 5 (B16 + Mock), n = 5 (B16 + CAR), n = 4 (B16-mCD19 + Mock), and n = 6 (B16-mCD19 + CAR).

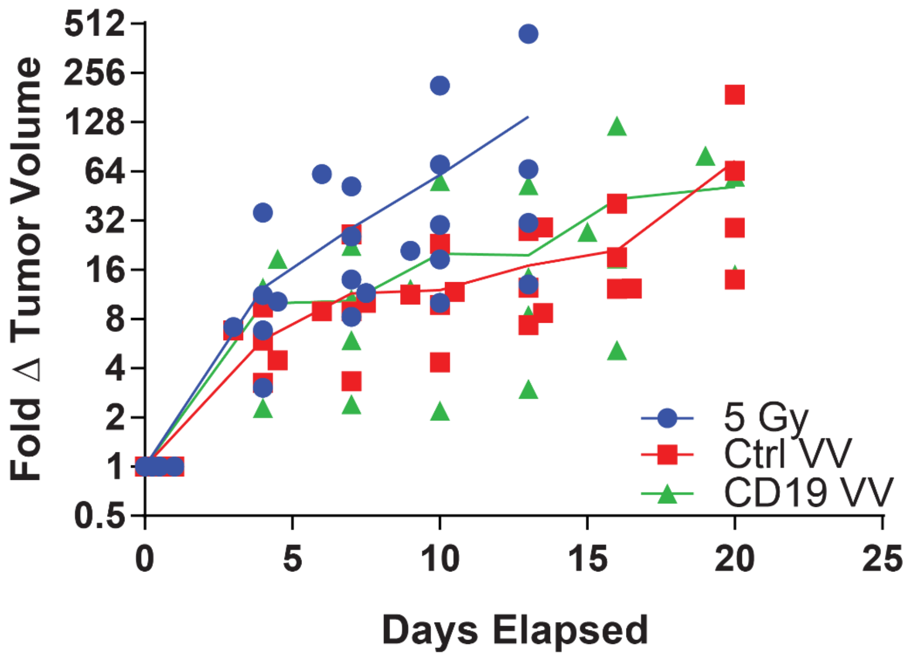


**Supplementary Figure S3. Therapeutic index of vaccinia viruses as single agents.** Intratumoral Control VV (n = 5 independent mice) and mCD19 VV (n = 4 independent mice) combined with TBI can yield modest delays in B16 tumor progression relative to mice only receiving TBI (n = 6 independent mice). <u>TBI Only vs. Control VV</u>: day 11 tumor volume p = 0.0040, t = 3.836, df = 9. <u>TBI Only vs. CD19 VV</u>: day 11 tumor volume p = 0.0246, t = 2.762, df = 8. The two viruses also have a similar therapeutic index *in vivo* <u>Control VV vs. CD19 VV</u>: p = 0.7293, t = 0.3602, df = 7.

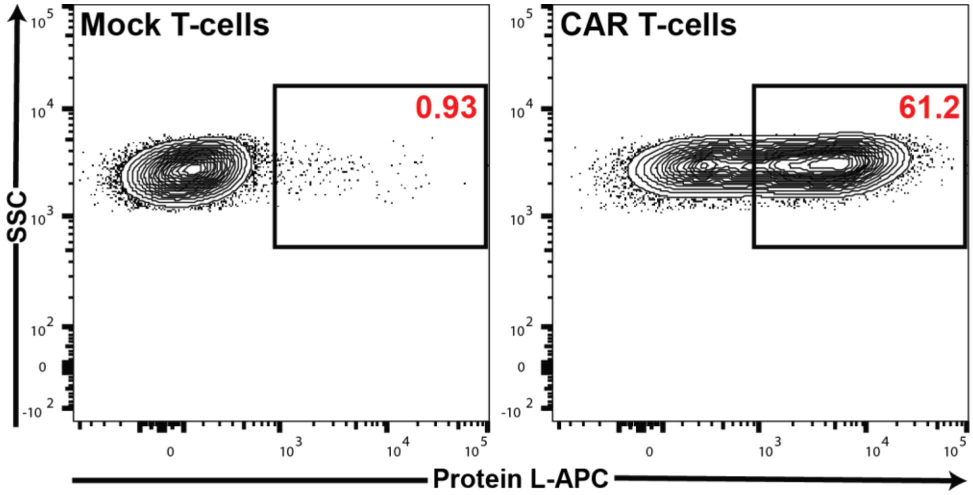


**Supplementary Figure S4. Generation of murine mCD19 CAR T-cells.**
Retroviral transduction of primary murine T-cell achieved efficiencies of ~50-65% and can be measured by staining chimeric antigen receptors with Protein L.
