# Supplementary material for: Viral Delivery of CAR Targets to Solid Tumors Enables Effective Cell Therapy
Source: Mol Ther Oncolytics. 2020 Apr 7;17:232–40. doi: 10.1016/j.omto.2020.03.018 (PMC7183102; doi:10.1016/j.omto.2020.03.018)
Supplement: Document S2. Article plus Supplemental Information [file mmc2.pdf]

Molecular Therapy
Oncolytics
Original Article

Viral Delivery of CAR Targets to Solid Tumors Enables Effective Cell Therapy

Amin Aalipour,[1,2] Fabrice Le Boeuf,[3,4] Matthew Tang,[3,4] Surya Murty,[1,2] Federico Simonetta,[5] Alexander X. Lozano,[6,7] Travis M. Shaffer,[2,8] John C. Bell,[3,4] and Sanjiv S. Gambhir[1,2,6,8,9]

[1]Department of Bioengineering, Stanford University School of Medicine, Stanford, CA 94305, USA; [2]Molecular Imaging Program at Stanford, Stanford University School of Medicine, Stanford, CA 94305, USA; [3]Cancer Therapeutics, Ottawa Hospital Research Institute, Ottawa, ON K1H 8L6, Canada; [4]Department of Biochemistry, Microbiology and Immunology, University of Ottawa, Ottawa, ON K1H 8M5, Canada; [5]Division of Blood and Marrow Transplantation, Department of Medicine, Stanford University School of Medicine, Stanford, CA 94305, USA; [6]Department of Materials Science and Engineering, Stanford University, Stanford, CA 94305, USA; [7]Faculty of Medicine, University of Toronto, Toronto, ON MS5 1A8, Canada; [8]Department of Radiology, Stanford University School of Medicine, Stanford, CA 94305, USA; [9]Canary Center at Stanford for Cancer Early Detection, Stanford University School of Medicine, Palo Alto, CA 94305, USA

Chimeric antigen receptor (CAR) T cell therapy has had limited efficacy for solid tumors, largely due to a lack of selectively and highly expressed surface antigens. To avoid reliance on a tumor's endogenous antigens, here we describe a method of tumor-selective delivery of surface antigens using an oncolytic virus to enable a generalizable CAR T cell therapy. Using CD19 as our proof of concept, we engineered a thymidine kinase-disrupted vaccinia virus to selectively deliver CD19 to malignant cells, and thus demonstrated potentiation of CD19 CAR T cell activity against two tumor types *in vitro*. In an immunocompetent model of B16 melanoma, this combination markedly delayed tumor growth and improved median survival compared with antigen-mismatched combinations. We also found that CD19 delivery could improve CAR T cell activity against tumor cells that express low levels of cognate antigen, suggesting a potential application in counteracting antigen-low escape. This approach highlights the potential of engineering tumors for effective adoptive cell therapy.

INTRODUCTION

Chimeric antigen receptor (CAR) T cell therapy has emerged as a promising curative cancer immunotherapy, with CD19-targeting CAR T cells having achieved durable remissions in the setting of therapy-resistant B cell malignancies.[1] Translating these successes to solid tumors is arguably the most pressing challenge facing the field.[2]

Solid tumors present several challenges for effective CAR T cell therapy, which have limited the success of efforts to date. For one, solid tumors are often populated with myeloid-derived suppressor cells that contribute to an immunosuppressive, anti-inflammatory microenvironment that can inhibit T cell function and proliferation.[3] Second, although lineage-restricted antigens such as CD19 are uniformly expressed and B cell aplasia is a clinically manageable phenotype, identifying solid tumor CAR targets that are both uniformly and selectively expressed on malignant cells to prevent "off-tumor, on-target" toxicities has proven problematic.[4] Moreover, CAR T cell recognition of target cells is highly dependent on antigen density, suggesting that even solid tumors that do express tumor-associated targets may still be unresponsive to therapy should they express low levels of antigen.[5–7]

To date, efforts to overcome these challenges have primarily focused on CAR T cell potency and persistence. Combination therapies involving radiation,[8] oncolytic viruses,[9] and checkpoint blockade[10,11] have been proposed to circumvent the immunosuppressive effects of the tumor microenvironment, reverse T cell exhaustion, and prevent antigen escape. Genetic engineering has also been used to disrupt negative regulators of T cell activation[12] or "armor" T cells with activation-sustaining cytokines.[13]

But despite novel methods of improving CAR T cell function, little effort has been dedicated to engineering tumors for effective adoptive cell therapy, and the lack of targetable surface antigens that are selectively expressed on malignant cells remains a major unaddressed challenge. This limitation also necessitates that new CARs be designed for each new proposed antigen. CAR T cells targeting putative solid tumor antigens, including Ganglioside G2 (GD2), mesothelin, B-cell maturation antige (BCMA), prostate-specific membrane antigen (PSMA), Epidermal Growth Factor Receptor Variant III (EGFRvIII), Mucin 1 (MUC1), and New York Esophageal Squamous Cell Carcinoma-1 (NY-ESO-1), are all under active investigation.[4]

To overcome the challenge of relying on endogenous solid tumor antigens, here we describe a method of selectively delivering an ectopic CAR target antigen to malignant cells using an oncolytic virus to enable a potentially universal approach to solid tumor CAR T cell therapy. Oncolytic viruses are ideal partners in this regard for the following reasons: (1) they can selectively infect and/or replicate in malignant cells to minimize delivery of the CAR target to healthy

Received 25 March 2020; accepted 27 March 2020;
https://doi.org/10.1016/j.omto.2020.03.018.

Correspondence: Sanjiv S. Gambhir, Department of Bioengineering, Stanford University School of Medicine, The James H. Clark Center, 318 Campus Drive, Room E150A, Stanford, CA 94305, USA.
E-mail: sgambhir@stanford.edu

 © 2020 The Author(s).
This is an open access article under the CC BY license (http://creativecommons.org/licenses/by/4.0/).

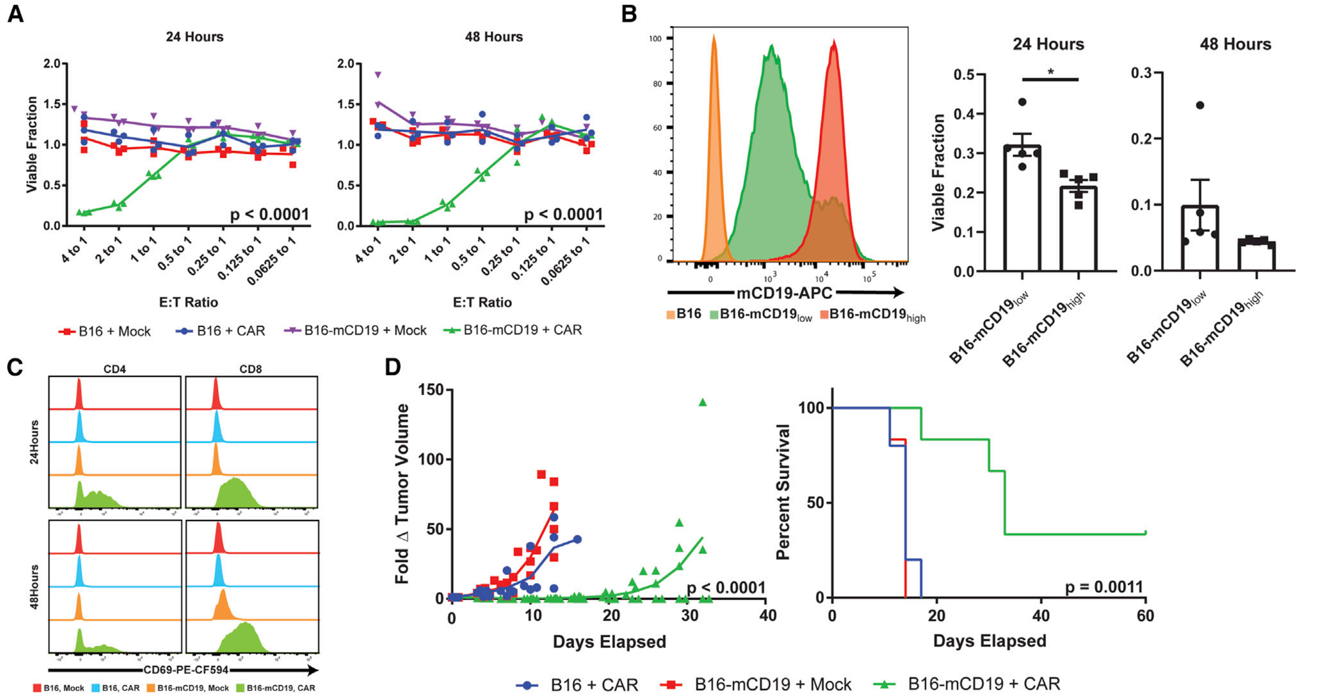

Figure 1. mCD19 CAR T Cells Exhibit Cytotoxic Activity against a B16-mCD19 Cell Line

(A) Dose- and time-dependent cytotoxicity of mCD19 CAR T cells and mock T cells in *in vitro* co-cultures against either native B16 cells or a B16 cell line engineered to express mCD19. 24-h E:T = 4, $p < 0.0001$, $F = 49.23$, $R^2 = 0.9486$ by ANOVA; 48-h E:T = 4, $p < 0.0001$, $F = 49.65$, $R^2 = 0.9490$ by ANOVA. n = 3 independent cultures for each combination, E:T ratio, and time point. (B) Antigen density-dependent mCD19 CAR T cell cytotoxicity against low- and high-mCD19-expressing B16 cell lines at 24 and 48 h of co-culture (n = 5 independent cultures with each cell line, $p = 0.0116$, $t = 3.258$, degrees of freedom (df) = 8 by two-tailed unpaired t test). (C) CD69 is upregulated only in antigen-matched co-cultures for both CD4 and CD8 T cells. (D) mCD19 CAR T cells significantly delay B16-mCD19 tumor progression *in vivo* (left) and confer a survival benefit relative to antigen-mismatched therapy groups. Day 8 tumor volume: $p < 0.0001$, $F = 19.14$, $R^2 = 0.7322$ by ANOVA. Kaplan-Meier survival curve: $p = 0.0011$, df = 2, chi-square = 13.58 by Mantel-Cox test. Number of independent mice in each group is as follows: n = 5 (B16 + CAR), n = 6 (B16-mCD19 + mock), and n = 6 (B16-mCD19 + CAR). Data are shown as mean ± standard error of the mean (SEM). Asterisks indicate statistical significance: *$p < 0.05$.

tissues;[14] (2) they have been shown to reprogram the immunosuppressive microenvironment of solid tumors into one that is more conducive to T cell activity;[15,16] (3) they have the capacity to selectively lyse infected cells and recruit the endogenous anti-viral immune response;[17] and (4) they have already demonstrated safety and efficacy in clinical trials, leading to US Food and Drug Administration (FDA) approval (talimogene laherparepvec in 2015).[18]

In our proof of concept, we designed a thymidine kinase-disrupted (TK^-) oncolytic vaccinia virus (VV) to selectively induce CD19 expression on malignant cells and thereafter treated the tumors with CD19-targeted CAR T cells. This method represents an important conceptual advance in a tumor-centric approach to CAR T cell therapy and could potentially enable a universal approach to solid tumor CAR T cell therapy that is agnostic to a tumor's native surface expression profile.

RESULTS

mCD19 CAR T Cells Exhibit Activity against mCD19-Positive Melanoma Cells

To characterize the activity of primary murine CD19 (mCD19) CAR T cells against solid tumors that uniformly express mCD19, we first stably expressed mCD19 and a TurboRFP/*Renilla* luciferase (TR) fusion protein in the B16 mouse melanoma cell line (Figure S1). Second generation murine mCD19 CAR T cells containing CD3ζ and CD28 costimulatory domains[19] exhibited potent cytotoxicity against the engineered B16-TR-mCD19 cell line in co-culture assays at effector-to-tumor (E:T) ratios of 0.5 and higher as measured by bioluminescence imaging ($p < 0.0001$; n = 3 for each condition) (Figure 1A). mCD19-negative B16 viability was not affected even at an E:T ratio of 4:1. Mock T cells, which were similarly activated with interleukin-2 (IL-2), IL-7, and anti-CD3/CD28 activation beads in culture, but not transduced with the mCD19 CAR, also lacked activity against either mCD19-positive or -negative B16 cells. CD19 CAR T cell toxicity was also dependent on antigen density, with a B16-mCD19$_{low}$ cell line exhibiting a diminished response compared with a B16-mCD19$_{high}$ cell line ($p = 0.0116$; n = 5 for each condition) (Figure 1B). Antigen-specific T cell cytotoxicity was confirmed by upregulation of the early T cell activation marker CD69 on both CD4 and CD8 T cells in only the properly matched B16-mCD19 + mCD19 CAR T cell condition (Figure 1C).

To assess the solid tumor activity of mCD19 CAR T cells *in vivo*, we established orthotopic, syngeneic models of B16 and B16-mCD19

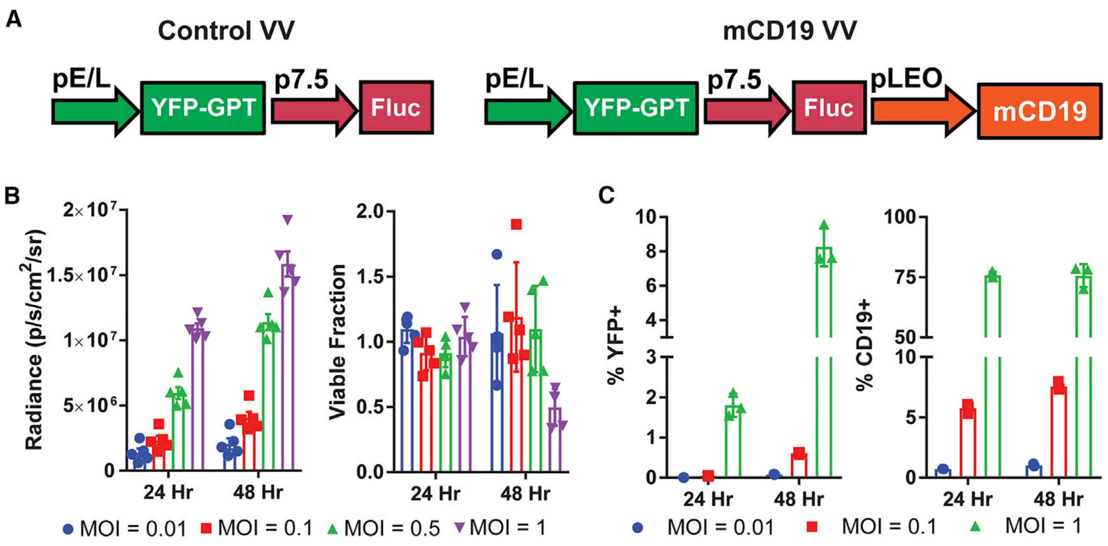

Figure 2. Design and Validation of Recombinant Vaccinia Viruses

(A) Design of Ctrl and mCD19 oncolytic vaccinia viruses (VVs). (B) Time- and dose-dependent expression of Fluc (left) and lytic activity (right) in B16 cells after infection with mCD19 VV. (C) Time- and dose-dependent expression of YFP and mCD19 in B16 cells infected with mCD19 VV. Data are shown as mean ± SEM. GPT, guanine phosphoribosyltransferase; p7.5, vaccinia 7.5-kDa early promoter; pE/L, early/late promoter; pLEO, synthetic late-early optimized promoter.

melanomas and treated the tumors with either intratumoral mCD19 CAR T cells or mock T cells immediately following a sub-lethal lymphodepletive regimen of 5 Gy total body irradiation (TBI) previously shown to be necessary for CD19 CAR activity in immunocompetent models.[19] Similar to the *in vitro* findings, the antigen-matched therapy group exhibited delayed tumor growth in all mice and completely eliminated the tumors in 33% of the mice ($p < 0.0001$; B16 + CAR: n = 5, B16-mCD19 + mock: n = 6, B16-mCD19 + CAR: n = 6) (Figure 1D). A single intravenous injection of CAR T cells was not an effective therapeutic approach even for antigen-positive tumors (Figure S2). Together, the data suggest that mCD19 CAR T cells can exhibit potent activity in solid tumors engineered to express ectopic mCD19.

Recombinant VV Can Deliver mCD19 to Malignant Cells

In order to selectively express an ectopic surface protein to malignant cells, we generated recombinant VVs with transgenes inserted into the viral TK locus. TK-disrupted VV is reliant on cellular TK for replication and can selectively propagate in tumor cells given their higher rates of nucleotide turnover.[20] We designed both a control (Ctrl) oncolytic VV (Ctrl VV) expressing firefly luciferase (Fluc) and yellow fluorescent protein (YFP),[21] as well as a version also encoding for mCD19 (mCD19 VV) (Figure 2A). Efficient VV replication in B16 cells was confirmed by time- and dose-dependent expression of Fluc, YFP, and mCD19 (Figures 2B and 2C), with up to 75% of cells expressing mCD19 at 48 h of culture with virus at a multiplicity of infection (MOI) of 1. Despite detectable transgene expression, the oncolytic virus did not induce significant cell death at an MOI of 0.01 or 0.1, highlighting the therapeutic limits of oncolytic virotherapy as a single agent (Figure 2B).

Infection with mCD19 VV Enables Antigen-Specific mCD19 CAR T Cell Activity

We next aimed to show that oncolytic virus-driven expression of ectopic CAR targets could enable selective killing by antigen-matched CAR T cells (Figure 3A). Cultured B16 cells were infected with Ctrl or mCD19 VV at an MOI of 0.2 for 48 h prior to addition of either mCD19 CAR T cells or mock T cells. The toxicity profile of each type of VV or T cell as a monotherapy was also evaluated. In B16 co-cultures, the combination of mCD19 VV together with mCD19 CAR T cells exhibited the highest toxicity 24 and 48 h following addition of T cells (Figure 3B). This effect was also seen in the SB28 murine glioma cell line, suggesting the generalizability of the approach across tumor types. VV-mediated mCD19 delivery also augmented CAR T cell activity against the B16-mCD19$_{low}$ cell line, highlighting potential uses of this approach to "boost" levels of tumor-associated surface antigen prior to therapy or as a method of overcoming antigen-low resistance. Enhanced cytotoxicity was mirrored by selective upregulation of CD69 on both CD4 and CD8 T cells in the antigen-matched combination (Figure 3C). Flow cytometry of B16 cells remaining 24 or 48 h after addition of T cells further confirmed T cell-mediated killing (Figure 3D). In the absence of T cells, both Ctrl and mCD19 VV expectedly induced expression of mCD19 and/or YFP. Addition of mock T cells did not affect these profiles, and similarly YFP expression from Ctrl VV still persisted 48 h following addition of CAR T cells. Notably, CAR T cells eliminated all mCD19$^+$ and YFP$^+$ cells by 48 h after co-culture with mCD19 VV-infected cells.

Tumor-Selective Delivery of mCD19 Potentiates mCD19 CAR T Cell Killing *In Vivo*

To assess our ability to force tumor expression of mCD19 *in vivo*, we injected orthotopic B16-TR tumors with three doses of 10^8 plaque-

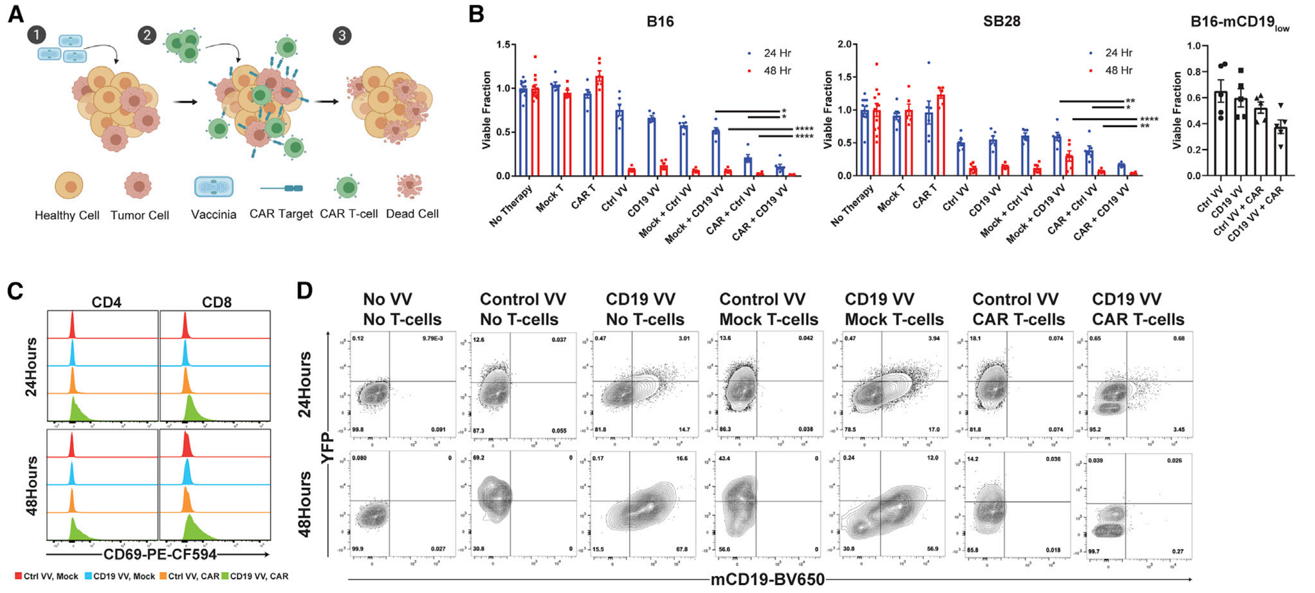

Figure 3. mCD19 CAR T Cells Eliminate mCD19 VV-Infected Tumor Cells *In Vitro*

(A) Schematic of tumor-selective delivery of CAR targets by an oncolytic VV followed by selective clearance by antigen-matched CAR T cells. (B) *In vitro* co-culture studies with B16 (left) and SB28 (middle) highlight the greatest combinatorial toxicity with co-culture of mCD19 CAR T cells and tumor cells infected with mCD19 VV. Mock + CD19 VV versus CAR + CD19 VV: $p < 0.0001$, $t = 9.413$, $df = 10$ (B16, 24 h); $p = 0.0009$; $t = 4.654$, $df = 10$ (B16, 48 h); $p < 0.0001$, $t = 6.993$, $df = 10$ (SB28, 24 h); $p = 0.0073$, $t = 3.356$, $df = 10$ (SB28 48 h). CAR + Ctrl VV versus CAR + CD19 VV: $p = 0.0272$, $t = 2.584$, $df = 10$ (B16, 24 h); $p = 0.0337$, $t = 2.459$, $df = 10$ (B16, 48 h); $p = 0.0076$, $t = 3.332$, $df = 10$ (SB28, 24 h); $p = 0.0377$, $t = 2.395$, $df = 10$ (SB28, 48 h). n = 6 independent cultures for each combination, time point, and cell line. CAR T cell efficacy at 24 h of co-culture benefits from VV-induced augmentation in levels of mCD19 in a B16-mCD19 cell line that expresses low levels of mCD19 (right). CAR + Ctrl VV versus CAR + CD19 VV: n = 5 independent cultures for each combination, $p = 0.0618$, $t = 2.170$, $df = 8$. (C) Expression of the early activation marker CD69 is upregulated in the antigen-matched combination of mCD19 VV-infected B16 cells and mCD19 CAR T cells in *in vitro* co-culture. (D) The presence of CAR T cells eliminates all mCD19$^+$ cells by 48 h of co-culture with mCD19 VV-infected B16 cells. Populations are shown gated on CD4$^-$CD8$^-$ double-negative cells. All statistical analyses were performed with unpaired two-tailed t tests. Data are shown as mean ± SEM. Asterisks indicate statistical significance: *$p < 0.05$, **$p < 0.01$, ****$p < 0.0001$.

forming units (PFUs) of mCD19 VV (days 1, 3, and 5). Doses were separated by 48 h, and YFP and mCD19 expression on TR$^+$ cells from resected tumors were measured 1 day following administration of the final viral dose (day 6). Without any lymphodepletive regimen, we observed, on average, 24% mCD19$^+$ and 14% YFP$^+$ cells within the TR$^+$ population (Figure 4A). Expectedly, rates of intratumoral T cell infection were markedly lower (T cell versus B16-TR %CD19$^+$, $p = 0.0042$ for 0 Gy and $p = 0.0014$ for 5 Gy), highlighting the tumor selectivity of TK-deleted VV and minimizing concerns of potential CAR T cell fratricide.

Oncolytic virotherapy as a single agent benefits from both direct lysis of infected tumor cells and recruitment of the endogenous anti-viral response. Although beneficial, this endogenous immune response can also impair viral propagation.[22] Because patients receiving CAR T cell therapy undergo lymphodepletive regimens of chemotherapy or radiation to enable expansion of the adoptively transferred cells, we hypothesized that this lymphopenia may also serve an added benefit of allowing enhanced oncolytic virus spread throughout a tumor. Consistent with this hypothesis, we observed that lymphodepletion with 5 Gy TBI immediately preceding administration of the first viral dose (n = 2) markedly increased the proportion of mCD19$^+$ (62%; $p = 0.0144$) and YFP$^+$ (34%; $p = 0.008$) cells in the TR$^+$ population compared with the no TBI group (n = 3). In this same model, we observed no significant differences in *in vivo* anti-tumor efficacy by two-tailed unpaired t test between the Ctrl (n = 5) and mCD19 (n = 4) VV, and both achieved modest delays (day 11: tumor volume Ctrl VV versus TBI only, $p = 0.004$; mCD19 VV versus TBI only, $p = 0.0246$) in tumor growth relative to the TBI-only group (n = 6) (Figure S3).

Encouraged by the effective *in vivo* delivery of mCD19 and motivated by the limited anti-tumor efficacy of the oncolytic virus as a monotherapy, we finally examined whether mCD19 CAR T cells could be redirected to engage with B16 cells infected with mCD19 VV *in vivo*. VV was again administered as a three-dose regimen (days 1, 3, and 5), and mice were treated with 5 Gy TBI and intratumoral injection of T cells on the same day as the first virus injection (day 1). All combinations of VV (Ctrl or mCD19) and T cells (mock or CAR) were evaluated. Analysis of intratumoral T cells 2 days following the final dose of VV (day 7) showed a substantial upregulation of the activation marker OX40 on both CD4 and CD8 T cells in the antigen-matched combination therapy relative to the other treatment groups (Figure 4B). Consistent with *ex vivo* results, the antigen-matched combination also achieved a significant (CAR + Ctrl VV [n = 4] versus CAR + CD19 VV [n = 5] day 11 tumor volume; $p = 0.0051$) delay

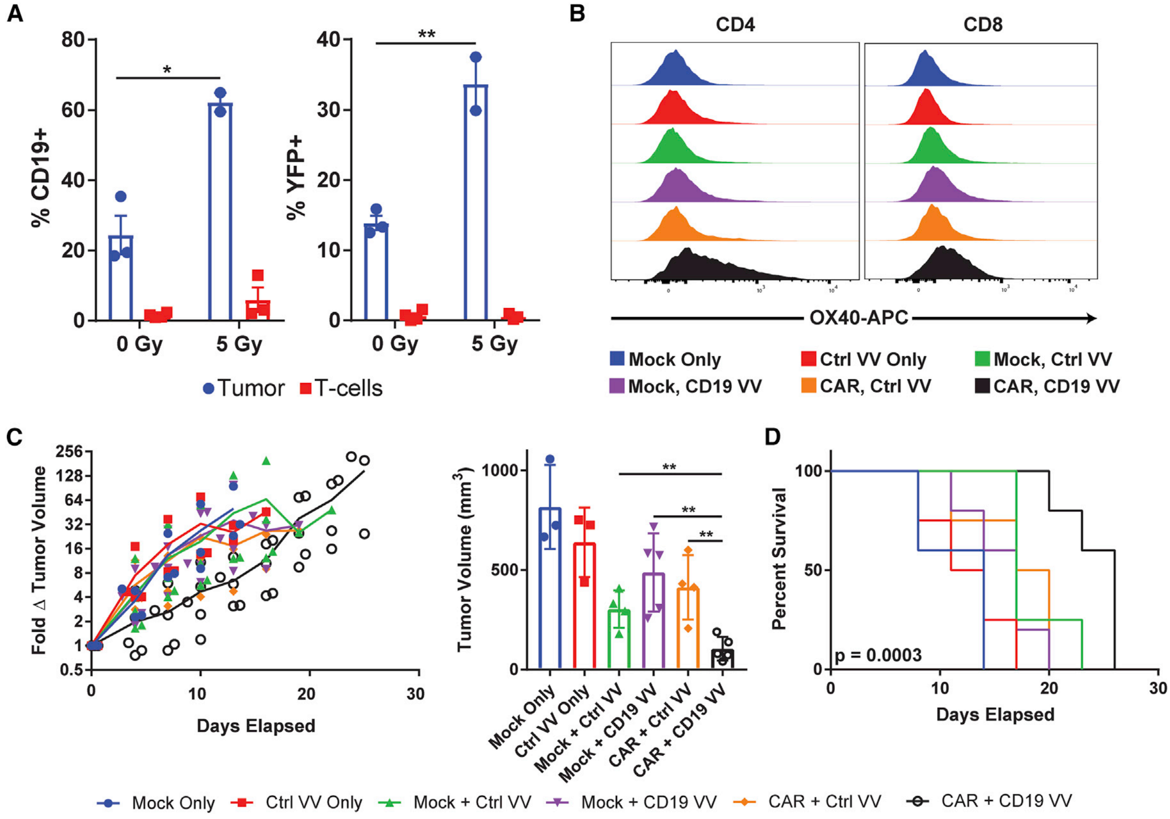

Figure 4. Orthotopic Melanoma Tumors Respond to Combination Therapy *In Vivo*

(A) Expression of mCD19 and YFP on B16 cells and tumor-infiltrating T cells in tumors injected intratumorally with CD19 VV. Rates of infection can be increased with a single dose of 5 Gy TBI on the day of the first virus injection. %mCD19 + TBI (n = 2 independent mice) versus no TBI (n = 3 independent mice): p = 0.0144, t = 5.126, df = 3. %YFP + TBI versus no TBI: p = 0.008, t = 6.332, df = 3. (B) Intratumoral CD4 and CD8 T cells upregulate the activation marker OX40 in the antigen-matched combination group. (C) The combination of mCD19 VV with mCD19 CAR T cells significantly delays B16 tumor progression. Fold change in tumor volume (left) and tumor volume on day 11 (right) are shown because this was the last day where all groups had ≥3 mice alive. Mock + Ctrl VV (n = 4 independent mice) versus CAR + CD19 VV (n = 5 independent mice) day 11 tumor volume: p = 0.006, t = 3.885, df = 7; mock + CD19 VV (n = 5 independent mice) versus CAR + CD19 VV day 11 tumor volume: p = 0.0031, t = 4.183, df = 8; CAR + Ctrl VV (n = 4 independent mice) versus CAR + CD19 VV day 11 tumor volume: p = 0.0051, t = 4.006, df = 7. (D) Survival curves for each combination therapy showing significant prolongation in the antigen-matched therapy. Kaplan-Meier survival curve: p = 0.0003, df = 5, chi-square = 23.33 by Mantel-Cox test. Number of independent mice in each group is as follows: n = 4 (mock only), n = 4 (Ctrl VV only), n = 5 (mock + Ctrl VV), n = 5 (mock + CD19 VV), n = 4 (CAR + Ctrl VV), n = 5 (CAR + CD19 VV). Non-survival statistical analysis was performed with unpaired two-tailed t tests. Data are shown as mean ± SEM (A) and mean ± standard deviation (C). Asterisks indicate statistical significance: *p < 0.05, **p < 0.01.

in tumor progression observable as early as 4 days following initiation of therapy (Figure 4C). This translated to a ~50% increase in median survival following therapy from 17 (mock + CD19 VV) and 18.5 days (CAR + Ctrl VV) to 26 days (CAR + CD19 VV) (Figure 4D).

DISCUSSION

Motivated by the lack of solid tumor surface antigens that can be efficiently targeted with CAR T cells, here we described a method of tumor-selective delivery of CAR targets using an oncolytic virus to enable a potentially universal approach to adoptive cell therapy. Using CD19 as a model antigen, we showed both *ex vivo* and *in vivo* that TK^{-} VV can selectively deliver a surface antigen, which can then be targeted by cognate CAR T cells with high specificity. We further demonstrated *in vitro* that the approach is generalizable to two different cell lines and can be effective for both tumors that do not express the ectopic target or express it at low levels.

Many features of our approach are modular and warrant further investigation. Although we demonstrated a proof of concept using TK-deleted VV given its large packaging capacity (genome size ~190 kb), ease of genetic manipulation, high immunogenicity,[23] and clinical translatability,[24] alternate oncolytic viruses (in particular RNA viruses with higher replicative rates, FDA-approved agents such as herpes viruses, or polio/measles viruses with the ability to cross the blood-brain barrier) could also be adapted as antigen-delivery vehicles.[25] This is an important consideration given potential variability in tumor susceptibility to vaccinia infection, suggesting that careful choice of vector may be necessary for each targeted tumor type. In addition, although the TK-deleted VV exhibited only minimal

infection of non-tumor cells (T cells) in our model, other more tumor-selective stains, such as the double-deleted strain of vaccinia,[26] could further mitigate off-target replication. Lastly, although we successfully infected ~60% of tumor cells *in vivo* in lymphodepleted hosts, there is also potential for using multiple oncolytic viruses with alternate mechanisms of tumor selectivity in order to increase tumor coverage. For example, although VV has demonstrated impressive infectivity across tumor types,[27–29] using multiple oncolytic viruses may be particularly useful to provide adequate coverage for variability in tumor entry receptor expression. For example, cell entry receptors for adenovirus include human coxsackie adenovirus receptor (hCAR), vascular cell adhesion molecule 1 (VCAM1), and CD46; whereas for herpes simplex they include herpesvirus entry mediator (HVEM), nectin 1, and nectin 2; and for pox viruses they include glycosaminoglycans (GAGs) and entry fusion complex (EFC).[23]

The overall modest delays in tumor progression with the antigen-matched versus antigen-mismatched groups (~50% increase in median survival from 17 to 26 days) may underestimate the efficacy of the combination therapy. For one, the standardized 5 Gy lymphodepletive TBI dose necessary for engrafting of CAR T cells and intratumoral spread of the vaccinia likely also had an independent therapeutic effect (Figure S2), thereby decreasing the window with which an incremental benefit from the antigen-matched combination therapy could be observed. Moreover, even injection of Ctrl VV with mock T cells is likely to have a therapeutic effect from both direct viral lysis and cell-mediated anti-viral immunity, further narrowing this window. More dramatic effect sizes may be observed if the antigen-matched combination therapy is compared with non-therapy or chemotherapy.

Delivery of therapeutic agents to solid tumors also continues to be a limitation of many immunotherapies, and our approach is also hindered in this regard. Consistent with prior studies on the efficacy of CAR T cells against B16 melanomas,[30] we showed that intravenous delivery of antigen-matched CAR T cells achieved only modest anti-tumor efficacy even when paired with TBI and exogenous cytokine, a result that necessitated intratumoral delivery of CAR T cells. Although this route of delivery has been the most effective in clinical trials for both oncolytic viruses[18,24] and CAR T cells,[31] further work is necessary to achieve efficient intravenous delivery of both agents to treat disseminated disease. Indeed, oncolytic viruses have been shown[32] to reshape the tumor microenvironment and promote recruitment of adoptively transferred T cells, and thus may be ideal partners in this regard.

There is also evidence that CAR T cells can induce epitope spreading and prime the endogenous anti-tumor response against otherwise non-immunogenic tumor neoantigens.[13,33] Future work will investigate whether the strategy described here can recapitulate this effect and trigger anti-tumor responses at non-treated tumor foci. This would in theory mitigate the need for viral infection of every malignant cell and CAR T cell delivery to all neoplastic lesions. CAR T cell therapy is thought to be uniquely well equipped to induce epitope via secretion of large quantities of interferon γ (IFNγ) and expression of high levels of CD40 ligand (CD40L),[34] both of which are key to recruiting and activating antigen-presenting cells. As such, the combination therapy may achieve a degree of epitope spreading not achievable with viral monotherapy alone.

Lastly, although we use CD19 in our proof of concept given that the CD19 CAR is the most clinically advanced, future iterations will employ surface antigens and cognate CARs that are not expressed on healthy tissue to avoid unnecessary B cell aplasia. Examples include viral surface proteins not present in mammalian hosts or mammalian proteins that are expressed only embryonically, such as placental alkaline phosphatase. Truncated cell surface proteins that lack intracellular domains can also be considered in future iterations to minimize potential signaling that could occur with expression of an ectopic receptor. Regardless, we envision this approach to be first used to deliver existing CAR targets in solid tumors to augment antigen levels prior to therapy, or in the setting of antigen-low acquired resistance. Boosting existing antigen levels could be a particularly useful therapeutic indication because CAR T cells are notorious for requiring high antigen density for activation (~10,000 antigens/cell versus ~10 peptides/cell for native T cells).[6] In this manner, we are optimistic that tumor engineering will emerge as a complementary approach to immune engineering in adoptive cell therapy.

MATERIALS AND METHODS

Cell Lines

B16 murine melanoma and HeLa human cervical cancer cells were obtained from American Type Cell Collection (Manassas, VA, USA), U2OS was obtained as a gift from Dr. Tobias Meyer, and SB28 murine glioma was obtained as a gift from Dr. Hideho Okada (University of California, San Francisco, San Francisco, CA, USA). Cells were cultured in DMEM supplemented with 10% fetal bovine serum (FBS) and 1% antibacterial/antimycotic solution (Thermo Fisher, Waltham, MA, USA), and maintained in a humidified, 5% CO_2 incubator at 37°C. B16-TurboRFP/RLuc8 (*Renilla* Luciferase 8) and SB28-TurboRFP/RLuc8 cell lines were generated by lentiviral transduction followed by three rounds of sorting for the highest 2.5% of TurboRFP expressers. B16-mCD19 and B16-mCD19-TurboRFP/RLuc8 cell lines were generated by transfection with Lipofectamine 3000 (Thermo Fisher) and three rounds of sorting for the highest 2.5% of mCD19 expressers.

Murine T Cell Isolation and CAR T Cell Generation

Primary murine T cells were isolated from spleens of healthy 6- to 8-week-old C57BL/6J mice (The Jackson Laboratory, Bar Harbor, ME, USA) using the EasySep Mouse T Cell Isolation Kit (STEMCELL Technologies, Vancouver, BC, Canada) following manufacturer's instructions and activated for 24 h in RPMI supplemented with 10% FBS, 1% antibacterial/antimycotic solution, 50 μM 2-mercaptoethanol (Thermo Fisher), 10 ng/mL each of IL-2 and IL-7 (PeproTech, Rocky Hill, NJ, USA), and CD3/CD28 Mouse T cell Activation Dynabeads (Thermo Fisher) at a bead-to-cell ratio of 1:1. The mCD19 CAR retrovirus producer cell line was obtained as a gift from Dr. Crystal

Mackall (Stanford University, Stanford, CA, USA). Retrovirus encoding the second generation mCD19 CAR with CD3ζ and CD28 co-stimulatory domains was centrifuged for 3 h at 3,200 rpm on non-adherent six-well plates that had been coated overnight at 4°C with 24 μg of RetroNectin (Takara Bio, Kusatsu, Shiga Prefecture, Japan) in 2 mL PBS/well. Viral supernatant was then removed, and 1×10^6 naive T cells were added in 4 mL media/well. Mock T cells were maintained in identical activation conditions but were not transduced with the CAR vector. After 48 h of transduction, CD3/CD28 activation beads were removed, and both mock and transduced CAR T cells were transferred to fresh medium supplemented with 10 ng/mL each of IL-2 and IL-7. Transduction efficiency (generally 50%–60%) was measured by Protein L staining (Figure S4) (Thermo Fisher) 24 h thereafter, and cells were used on the same day. Administered doses of CAR T cells were normalized based on transduction efficiency, and an equivalent number of total T cells were used in mock T cell conditions.

Recombinant VV Generation

mCD19 under control of the pLEO (synthetic late-early optimized promoter)[35] was cloned into the previously described pSEM-1 vector[21] that expresses Fluc and a YFP/guanine phosphoribosyltransferase fusion protein (YFP/GPT) under control of pE/L (early/late promoter) and p7.5 (vaccinia 7.5-kDa early promoter), respectively. U2OS cells were infected with the VV Copenhagen strain at an MOI of 0.01 for 2 h and then transfected with plasmid pE/L-YFP/GPT-p7.5-Fluc-pLEO-mCD19 using Lipofectamine 2000 (Thermo Fisher). The cells were incubated at 37°C for 4 h, the medium was replaced, and cells were cultured for an additional 48 h. Viruses were released from the cells by three freeze-thaw cycles at −80°C. The harvested viruses were used to infect a monolayer of U2OS cells. Virus inoculum was removed from the cells after 1.5 h, and complete DMEM containing 10% FBS and 1.5% carboxymethylcellulose (CMC) was added. YFP-positive virus plaques were plaque purified for six rounds of selection using U2OS cells. The plaque-purified virus was subject to 36% sucrose cushion purification and resuspended in Tris-HCl (pH 9.0).

Vaccinia Propagation and Titer Determination

VVs were expanded in HeLa cells by infecting ~95% confluent flasks at an MOI of 0.1 and harvesting the cells by mechanical scraping 48 h thereafter. Harvested cells were pelleted, resuspended in 40 mL of 1 mM Tris-HCl (pH 9.0), and subjected to three freeze-thaw cycles at −80°C to lyse the cells. Resulting cell debris was removed by centrifugation, and the cleared lysate was subjected to sucrose cushion purification with a 36% sucrose cushion. Viral pellets were resuspended in 1 mM Tris-HCl (pH 9.0) and stored at −80°C.

Viruses were titered by plaque assay using U2OS cells. In brief, 3.33×10^6 cells were plated overnight in each well of a 12-well plate and infected with purified virus serially diluted in serum-free DMEM at 37°C. After 2 h, the virus was removed and replaced with complete DMEM containing 10% FBS and 1.5% CMC. After 3 days of incubation, the overlay was aspirated, and cells were washed once with PBS and stained with 1 mL of a 0.1% crystal violet solution in water (Sigma-Aldrich) for 10 min at room temperature. Cells were washed once with distilled water, and plaques were counted to determine viral titer.

Cytotoxicity Assays

To evaluate the cytotoxicity of CAR or mock T cells as single agents, we plated 2×10^4 B16-TurboRFP/RLuc8 or B16-TurboRFP/RLuc8-mCD19 cells in 100 μL of DMEM in black 96-well plates. The following day, T cells were added at specified E:T ratios based on the initial number of plated tumor cells, and a viable fraction of tumors cells was measured 24 and 48 h thereafter by comparing fractional bioluminescence signal from RLuc8 between treated and untreated wells. Imaging of RLuc8 was performed on an IVIS-50 system (PerkinElmer, Waltham, MA, USA) immediately after washing cells once with PBS and addition of 200 μL of 1 μg/mL coelenterazine (NanoLight Technologies, Pinetop, AZ, USA) to each well. Images were taken with an exposure time of 60 s, F-stop of 8, and medium binning.

In combination studies with both VV and T cells, B16-TurboRFP/RLuc8 or SB28-TurboRFP/RLuc cells were instead plated at 10^4 cells/well (to account for longer duration of experiment) in 100 μL DMEM. The following day, the media were removed and replaced with 150 μL DMEM containing either Ctrl or mCD19 VV at an MOI of 0.2 (B16), 0.05 (SB28), or 0.1 (B16-mCD19$_{low}$). After 48 h of infection, T cells were added at an E:T ratio of 4:1 (B16 and SB28) or 1:1 (B16-mCD19$_{low}$) relative to initial number of plated cells (given the time delay from initial cell plating), and viability was assayed as previously described.

Tumor Harvesting

Resected B16 tumors were digested in 4 mL Hank's balanced salt solution (HBSS) containing 10 μg/mL DNase I (Sigma-Aldrich) and 100 μg/mL Liberase TL (Roche, Basel, Switzerland) for 60 min at 37°C. The solution was then diluted with PBS, filtered through a 70-μm filter, and spun at 300 × *g* for 5 min. Cells were resuspended in 1 mL ACK lysis buffer (Thermo Fisher) on ice for 5 min, washed once with PBS, and finally resuspended in PBS for staining.

Flow Cytometry

Single-cell suspensions were first stained with LIVE/DEAD Fixable Aqua Dead Cell Stain Kit (Thermo Fisher) following the manufacturer's instructions and then resuspended for 10 min in 100 μL fluorescence-activated cell sorting (FACS) buffer (PBS + 2% FBS) with 1 μg/10^6 TruStain FcX Antibody (BioLegend, San Diego, CA, USA) to block Fc receptors. When measuring rates of *in vivo* infection, both live and dead tumor cells were included in the analysis to better estimate rates of transduction. Flow antibodies were used at a concentration of 0.2 μg/10^6 cells in 100 μL volume when the concentration was provided and following the manufacturer's instructions if the concentration was unspecified. The following antibodies were used for staining: BUV395 Hamster Anti-Mouse CD3e Clone 145-2C11 (CAT#563565; BD Biosciences, San Jose, CA, USA), PerCP/

Cyanine5.5 Rat Anti-Mouse CD4 Clone RM4-5 (CAT#100540; BioLegend), Brilliant Violet 650 Rat Anti-Mouse CD19 Clone 6D5 (CAT#115541; BioLegend), PE Rat Anti-Mouse CD19 Clone 6D5 (CAT#115508; BioLegend), APC Rat Anti-Mouse CD19 Clone 6D5 (CAT#115512; BioLegend), PE-CF594 Hamster Anti-Mouse CD69 Clone H1.2F3 (CAT#562455; BD Biosciences), and APC Rat Anti-Mouse OX40 Clone OX-86 (CAT#119414; BioLegend). Following staining for 20 min on ice, cells were washed once with PBS, fixed in 4% paraformaldehyde for 20 min on ice, washed twice with FACS buffer, and resuspended in FACS buffer for analysis on a LSRII analyzer (Becton Dickinson, Franklin Lakes, NJ, USA).

In Vivo Therapy Studies

All animal experiments were performed under a protocol approved by the Stanford University Administrative Panels on Laboratory Animal Care (APLAC) and conducted in accordance with ethical guidelines prescribed therein. Six- to eight-week-old female C57BL/6 were implanted subcutaneously on the right shoulder with between 5×10^5 and 10^6 B16 or B16-mCD19 cells in 50–75 μL PBS. Tumors were allowed to grow for, on average, 7 days, and therapy was initiated once tumors reached an average volume of 25–50 mm^3. Mice were irradiated with 5 Gy, administered 10^7 intratumoral CAR or mock T cells in 50 μL PBS, and injected intratumorally with 10^8 PFUs VV in 30 μL PBS on the first day of therapy. Vaccinia was administered again on days 3 and 5 for a total of three injections. Mice receiving T cells also received intraperitoneal injections of 10 μg recombinant human IL-2 (PeproTech, Rocky Hill, NJ, USA) twice daily for the first 3 days of the therapy. Tumor size was measured by caliper every third day, and mice were sacrificed if any dimension exceeded 15 mm or ulceration exceeded 0.5 cm^2.

Statistical Analysis

Statistical analysis was performed using ordinary one-way ANOVA or two-tailed unpaired t tests. Statistical differences in survival curves were determined using Mantel-Cox tests. All statistical analysis was performed in GraphPad Prism Version 8.0.2 (San Diego, CA, USA).

SUPPLEMENTAL INFORMATION

Supplemental Information can be found online at https://doi.org/10.1016/j.omto.2020.03.018.

AUTHOR CONTRIBUTIONS

A.A. and S.S.G. conceived of the project and designed all experiments. F.L.B., M.T., and J.C.B. contributed to the design and synthesis of recombinant VVs. A.A., S.M., F.S., A.X.L., and T.M.S. performed experiments. A.A. and S.S.G. wrote the manuscript with contributions from all authors.

CONFLICTS OF INTEREST

A.A. and S.S.G. are co-inventors of a provisional patent filed on the subject of this work.

ACKNOWLEDGMENTS

Funding for this work was provided by the Canary Foundation and the Ben and Catherine Ivey Foundation (S.S.G.). Data were collected on an instrument in the Stanford Shared FACS Facility obtained using NIH S10 Shared Instrument Grant S10RR027431-01. A.A. has received support from Stanford Medical Scientist Training Program grant T32 GM007365, National Cancer Institute grant F30 CA236339-01A1, the Paul and Daisy Soros Fellowship, and the Bio-X Graduate Student Fellowship.

REFERENCES

1. June, C.H., and Sadelain, M. (2018). Chimeric Antigen Receptor Therapy. N. Engl. J. Med. *379*, 64–73.
2. D'Aloia, M.M., Zizzari, I.G., Sacchetti, B., Pierelli, L., and Alimandi, M. (2018). CAR-T cells: the long and winding road to solid tumors. Cell Death Dis. *9*, 282.
3. Newick, K., Moon, E., and Albelda, S.M. (2016). Chimeric antigen receptor T-cell therapy for solid tumors. Mol. Ther. Oncolytics *3*, 16006.
4. Martinez, M., and Moon, E.K. (2019). CAR T Cells for Solid Tumors: New Strategies for Finding, Infiltrating, and Surviving in the Tumor Microenvironment. Front. Immunol. *10*, 128.
5. Mackall, C.L. (2017). Enhancing the Efficacy of CAR T Cells. Blood *130* (*Suppl 1*), SCI-15.
6. Walker, A.J., Majzner, R.G., Zhang, L., Wanhainen, K., Long, A.H., Nguyen, S.M., Lopomo, P., Vigny, M., Fry, T.J., Orentas, R.J., and Mackall, C.L. (2017). Tumor Antigen and Receptor Densities Regulate Efficacy of a Chimeric Antigen Receptor Targeting Anaplastic Lymphoma Kinase. Mol. Ther. *25*, 2189–2201.
7. Hamieh, M., Dobrin, A., Cabriolu, A., van der Stegen, S.J.C., Giavridis, T., Mansilla-Soto, J., Eyquem, J., Zhao, Z., Whitlock, B.M., Miele, M.M., et al. (2019). CAR T cell trogocytosis and cooperative killing regulate tumour antigen escape. Nature *568*, 112–116.
8. DeSelm, C., Palomba, M.L., Yahalom, J., Hamieh, M., Eyquem, J., Rajasekhar, V.K., and Sadelain, M. (2018). Low-Dose Radiation Conditioning Enables CAR T Cells to Mitigate Antigen Escape. Mol. Ther. *26*, 2542–2552.
9. Wing, A., Fajardo, C.A., Posey, A.D., Jr., Shaw, C., Da, T., Young, R.M., Alemany, R., June, C.H., and Guedan, S. (2018). Improving CART-Cell Therapy of Solid Tumors with Oncolytic Virus-Driven Production of a Bispecific T-cell Engager. Cancer Immunol. Res. *6*, 605–616.
10. Chong, E.A., Melenhorst, J.J., Lacey, S.F., Ambrose, D.E., Gonzalez, V., Levine, B.L., June, C.H., and Schuster, S.J. (2017). PD-1 blockade modulates chimeric antigen receptor (CAR)-modified T cells: refueling the CAR. Blood *129*, 1039–1041.
11. Cherkassky, L., Morello, A., Villena-Vargas, J., Feng, Y., Dimitrov, D.S., Jones, D.R., Sadelain, M., and Adusumilli, P.S. (2016). Human CAR T cells with cell-intrinsic PD-1 checkpoint blockade resist tumor-mediated inhibition. J. Clin. Invest. *126*, 3130–3144.
12. Rupp, L.J., Schumann, K., Roybal, K.T., Gate, R.E., Ye, C.J., Lim, W.A., and Marson, A. (2017). CRISPR/Cas9-mediated PD-1 disruption enhances anti-tumor efficacy of human chimeric antigen receptor T cells. Sci. Rep. *7*, 737.
13. Adachi, K., Kano, Y., Nagai, T., Okuyama, N., Sakoda, Y., and Tamada, K. (2018). IL-7 and CCL19 expression in CAR-T cells improves immune cell infiltration and CAR-T cell survival in the tumor. Nat. Biotechnol. *36*, 346–351.
14. Jhawar, S.R., Thandoni, A., Bommareddy, P.K., Hassan, S., Kohlhapp, F.J., Goyal, S., Schenkel, J.M., Silk, A.W., and Zloza, A. (2017). Oncolytic Viruses-Natural and Genetically Engineered Cancer Immunotherapies. Front. Oncol. *7*, 202.
15. Bourgeois-Daigneault, M.-C., Roy, D.G., Aitken, A.S., El Sayes, N., Martin, N.T., Varette, O., Falls, T., St-Germain, L.E., Pelin, A., Lichty, B.D., et al. (2018). Neoadjuvant oncolytic virotherapy before surgery sensitizes triple-negative breast cancer to immune checkpoint therapy. Sci. Transl. Med. *10*, eaao1641.
16. Samson, A., Scott, K.J., Taggart, D., West, E.J., Wilson, E., Nuovo, G.J., Thomson, S., Corns, R., Mathew, R.K., Fuller, M.J., et al. (2018). Intravenous delivery of oncolytic

reovirus to brain tumor patients immunologically primes for subsequent checkpoint blockade. Sci. Transl. Med. *10*, eaam7577.

17. Marelli, G., Howells, A., Lemoine, N.R., and Wang, Y. (2018). Oncolytic Viral Therapy and the Immune System: A Double-Edged Sword Against Cancer. Front. Immunol. *9*, 866.
18. Andtbacka, R.H.I., Kaufman, H.L., Collichio, F., Amatruda, T., Senzer, N., Chesney, J., Delman, K.A., Spitler, L.E., Puzanov, I., Agarwala, S.S., et al. (2015). Talimogene Laherparepvec Improves Durable Response Rate in Patients With Advanced Melanoma. J. Clin. Oncol. *33*, 2780–2788.
19. Kochenderfer, J.N., Yu, Z., Frasheri, D., Restifo, N.P., and Rosenberg, S.A. (2010). Adoptive transfer of syngeneic T cells transduced with a chimeric antigen receptor that recognizes murine CD19 can eradicate lymphoma and normal B cells. Blood *116*, 3875–3886.
20. Parato, K.A., Breitbach, C.J., Le Boeuf, F., Wang, J., Storbeck, C., Ilkow, C., Diallo, J.S., Falls, T., Burns, J., Garcia, V., et al. (2012). The oncolytic poxvirus JX-594 selectively replicates in and destroys cancer cells driven by genetic pathways commonly activated in cancers. Mol. Ther. *20*, 749–758.
21. Rintoul, J.L., Wang, J., Gammon, D.B., van Buuren, N.J., Garson, K., Jardine, K., Barry, M., Evans, D.H., and Bell, J.C. (2011). A selectable and excisable marker system for the rapid creation of recombinant poxviruses. PLoS ONE *6*, e24643.
22. Filley, A.C., and Dey, M. (2017). Immune System, Friend or Foe of Oncolytic Virotherapy? Front. Oncol. *7*, 106.
23. Bommareddy, P.K., Shettigar, M., and Kaufman, H.L. (2018). Integrating oncolytic viruses in combination cancer immunotherapy. Nat. Rev. Immunol. *18*, 498–513.
24. Park, B.-H., Hwang, T., Liu, T.-C., Sze, D.Y., Kim, J.-S., Kwon, H.-C., Oh, S.Y., Han, S.Y., Yoon, J.H., Hong, S.H., et al. (2008). Use of a targeted oncolytic poxvirus, JX-594, in patients with refractory primary or metastatic liver cancer: a phase I trial. Lancet Oncol. *9*, 533–542.
25. Lawler, S.E., Speranza, M.-C., Cho, C.-F., and Chiocca, E.A. (2017). Oncolytic Viruses in Cancer Treatment: A Review. JAMA Oncol. *3*, 841–849.
26. McCart, J.A., Ward, J.M., Lee, J., Hu, Y., Alexander, H.R., Libutti, S.K., Moss, B., and Bartlett, D.L. (2001). Systemic cancer therapy with a tumor-selective vaccinia virus mutant lacking thymidine kinase and vaccinia growth factor genes. Cancer Res. *61*, 8751–8757.
27. Deng, L., Fan, J., Ding, Y., Zhang, J., Zhou, B., Zhang, Y., and Huang, B. (2017). Oncolytic efficacy of thymidine kinase-deleted vaccinia virus strain Guang9. Oncotarget *8*, 40533–40543.
28. Whitbeck, J.C., Foo, C.-H., Ponce de Leon, M., Eisenberg, R.J., and Cohen, G.H. (2009). Vaccinia virus exhibits cell-type-dependent entry characteristics. Virology *385*, 383–391.
29. Sánchez-Puig, J.M., Sánchez, L., Roy, G., and Blasco, R. (2004). Susceptibility of different leukocyte cell types to Vaccinia virus infection. Virol. J. *1*, 10.
30. Hu, B., Ren, J., Luo, Y., Keith, B., Young, R.M., Scholler, J., Zhao, Y., and June, C.H. (2017). Augmentation of Antitumor Immunity by Human and Mouse CAR T Cells Secreting IL-18. Cell Rep. *20*, 3025–3033.
31. Adusumilli, P.S., Cherkassky, L., Villena-Vargas, J., Colovos, C., Servais, E., Plotkin, J., Jones, D.R., and Sadelain, M. (2014). Regional delivery of mesothelin-targeted CAR T cell therapy generates potent and long-lasting CD4-dependent tumor immunity. Sci. Transl. Med. *6*, 261ra151.
32. Tähtinen, S., Grönberg-Vähä-Koskela, S., Lumen, D., Merisalo-Soikkeli, M., Siurala, M., Airaksinen, A.J., Vähä-Koskela, M., and Hemminki, A. (2015). Adenovirus Improves the Efficacy of Adoptive T-cell Therapy by Recruiting Immune Cells to and Promoting Their Activity at the Tumor. Cancer Immunol. Res. *3*, 915–925.
33. Slaney, C.Y., von Scheidt, B., Davenport, A.J., Beavis, P.A., Westwood, J.A., Mardiana, S., Tscharke, D.C., Ellis, S., Prince, H.M., Trapani, J.A., et al. (2017). Dual-specific Chimeric Antigen Receptor T Cells and an Indirect Vaccine Eradicate a Variety of Large Solid Tumors in an Immunocompetent, Self-antigen Setting. Clin. Cancer Res. *23*, 2478–2490.
34. Heckler, M., and Dougan, S.K. (2018). Unmasking Pancreatic Cancer: Epitope Spreading After Single Antigen Chimeric Antigen Receptor T-Cell Therapy in a Human Phase I Trial. Gastroenterology *155*, 11–14.
35. Di Pilato, M., Mejías-Pérez, E., Gómez, C.E., Perdiguero, B., Sorzano, C.O.S., and Esteban, M. (2013). New vaccinia virus promoter as a potential candidate for future vaccines. J. Gen. Virol. *94*, 2771–2776.

OMTO, Volume *17*

Supplemental Information

Viral Delivery of CAR Targets to Solid Tumors Enables Effective Cell Therapy

Amin Aalipour, Fabrice Le Boeuf, Matthew Tang, Surya Murty, Federico Simonetta, Alexander X. Lozano, Travis M. Shaffer, John C. Bell, and Sanjiv S. Gambhir

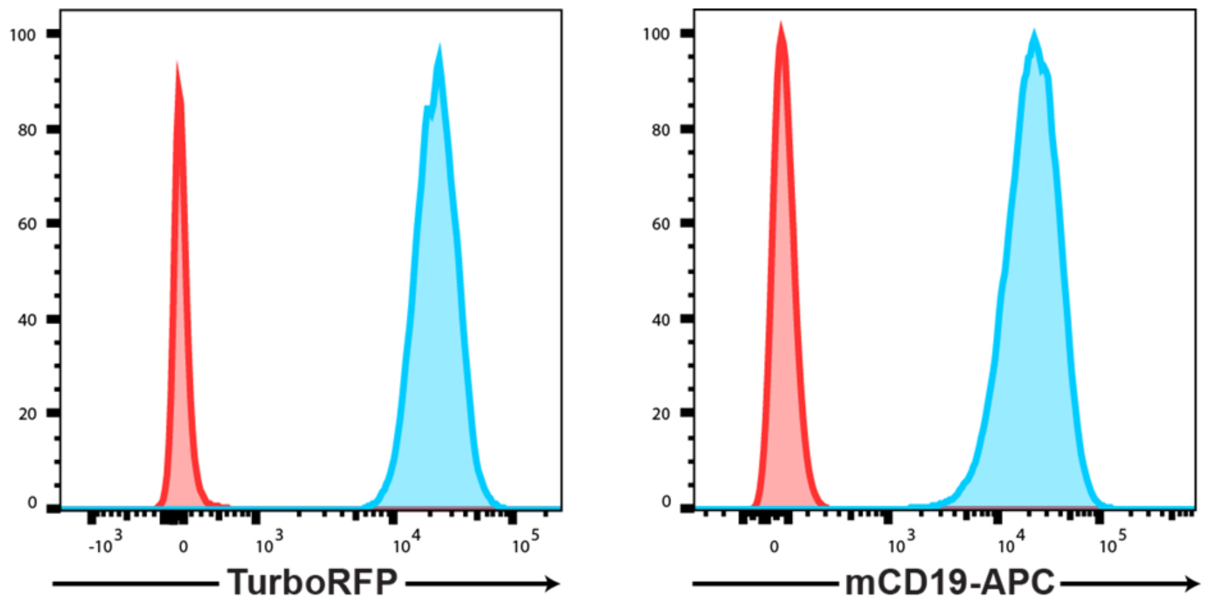

Supplementary Figure S1. Generation of engineered B16 cell lines.
Three cell lines were generated: B16-TurboRFP/RLuc8, B16-mCD19, and B16-TurboRFP/RLuc8-mCD19. Flow cytometry confirms uniform expression of TurboRFP (left) and mCD19 (right) transgenes. Data shown for B16-TurboRFP/RLuc8-mCD19 cell line (blue) relative to native B16 cell line (red).

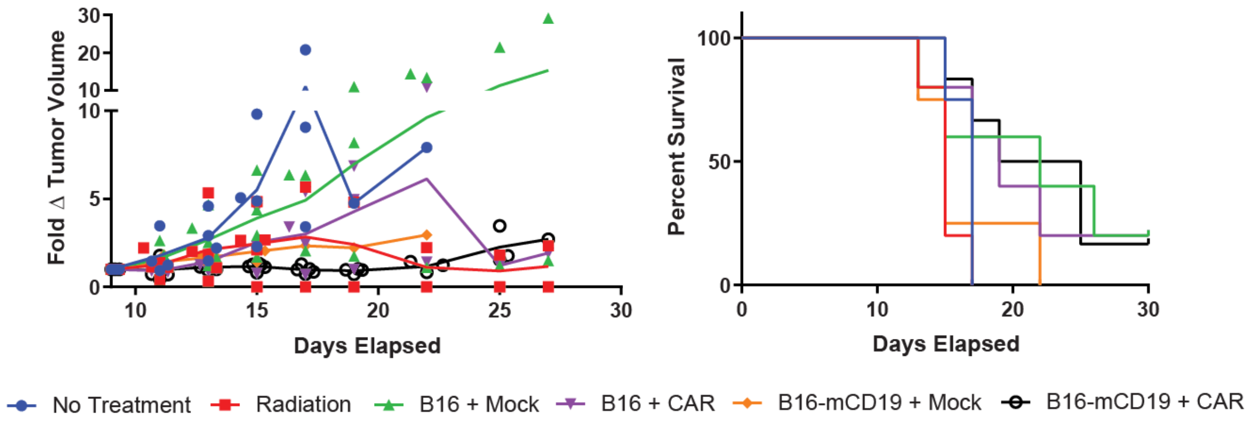

Supplementary Figure S2. Intravenous delivery of mCD19 CAR T-cells is ineffective against B16 tumors constitutively expressing mCD19.
Intravenously delivered mCD19 CAR T-cells do not significantly delay B16-mCD19 tumor progression *in vivo* (left) or confer a survival benefit (right) relative to tumors treated with 5Gy TBI (radiation), mock T-cells, or antigen negative tumors treated with either mock or CAR T-cells. Kaplan-Meier survival curve p = 0.0902, df = 5, Chi square = 9.514 by Mantel-Cox test. Number of independent mice in each group as follows: n = 4 (No Treatment), n = 5 (Radiation), n = 5 (B16 + Mock), n = 5 (B16 + CAR), n = 4 (B16-mCD19 + Mock), and n = 6 (B16-mCD19 + CAR).

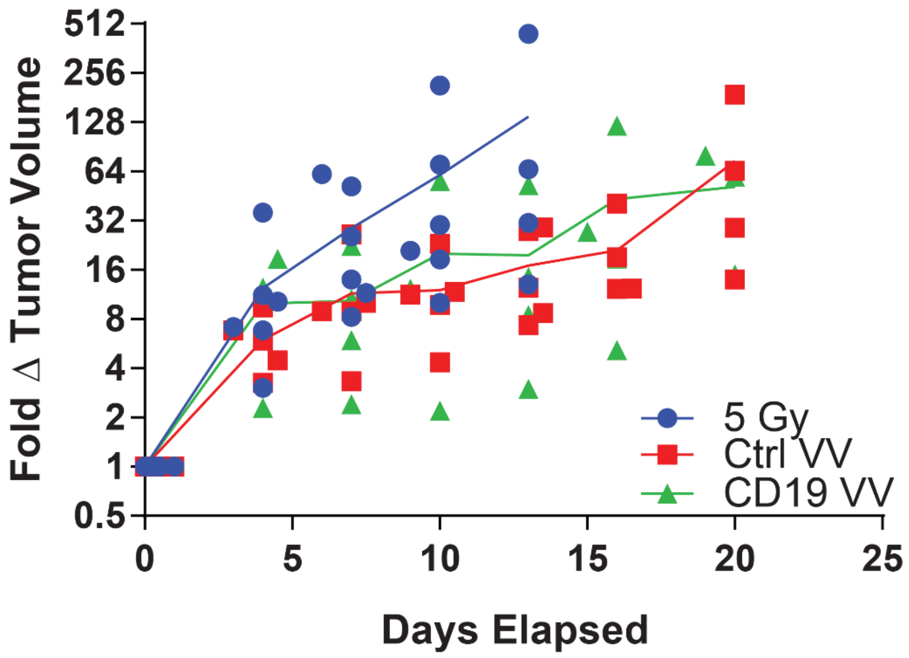

Supplementary Figure S3. Therapeutic index of vaccinia viruses as single agents. Intratumoral Control VV (n = 5 independent mice) and mCD19 VV (n = 4 independent mice) combined with TBI can yield modest delays in B16 tumor progression relative to mice only receiving TBI (n = 6 independent mice). <u>TBI Only vs. Control VV</u>: day 11 tumor volume p = 0.0040, t = 3.836, df = 9. <u>TBI Only vs. CD19 VV</u>: day 11 tumor volume p = 0.0246, t = 2.762, df = 8. The two viruses also have a similar therapeutic index *in vivo* <u>Control VV vs. CD19 VV</u>: p = 0.7293, t = 0.3602, df = 7.

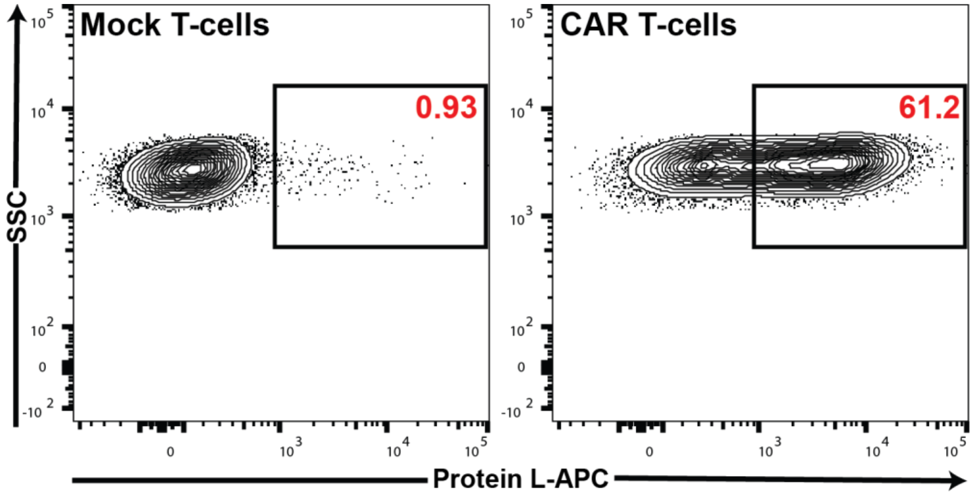

Supplementary Figure S4. Generation of murine mCD19 CAR T-cells.
Retroviral transduction of primary murine T-cell achieved efficiencies of ~50-65% and can be measured by staining chimeric antigen receptors with Protein L.
